# Associations of breastfeeding or formula feeding with infant anthropometry and body composition at 6 months

**DOI:** 10.1111/mcn.13105

**Published:** 2020-11-03

**Authors:** Muna J. Tahir, Keisuke Ejima, Peng Li, Ellen W. Demerath, David B. Allison, David A. Fields

**Affiliations:** ^1^ Division of Epidemiology and Community Health University of Minnesota Minneapolis Minnesota USA; ^2^ Department of Epidemiology and Biostatistics Indiana University School of Public Health‐Bloomington Bloomington Indiana USA; ^3^ Institute of Industrial Science The University of Tokyo Tokyo Japan; ^4^ Department of Biostatistics University of Alabama at Birmingham Birmingham Alabama USA; ^5^ School of Nursing University of Alabama at Birmingham Birmingham Alabama USA; ^6^ Department of Pediatrics University of Oklahoma Health Sciences Center Oklahoma City Oklahoma USA

**Keywords:** anthropometry, body composition, breastfeeding, childhood obesity, early growth, infant formula

## Abstract

The objective of this study was to investigate the associations of mode of feeding with infant anthropometric and body composition variables at 6 months of age. We studied 259 infants whose exclusive mode of feeding (breast or formula) to 1 month was confirmed. Standard anthropometric characteristics of the infants (weight, length and weight‐for‐length *z* scores) were obtained, and body composition (total fat mass, fat‐free mass, trunk fat mass and body fat percent) was measured using dual‐energy X‐ray absorptiometry (DXA) at 6 months (±12 days). General linear models were used to test the associations of mode of feeding with infant anthropometric and body composition variables at 6 months after adjustment for maternal and infant covariates. In this cohort of predominantly breastfed, White infants of highly educated mothers, fat‐free mass was lower (*P* = .002), and trunk fat mass (*P* = .032) and body fat percent (*P* < .001) were greater in breastfed infants than in formula‐fed infants at 6 months of age. After adjustment for covariates, total fat‐free mass was significantly lower (*β* = −372 g, [SE = 125, *P =* .003]), and body fat percent was significantly greater (*β* = 3.30, [SE = 0.91, *P* < .001]) in breastfed infants than in formula‐fed infants. No other significant associations were observed. These findings support those of previous studies reporting greater fat‐free mass in formula‐fed infants during the first 6 months of life. Additional research is warranted to explore whether differences in infant body composition by mode of feeding persist throughout the life course and to assess causality.

**Key messages**

- The role of breastfeeding in influencing offspring body composition remains unsettled.
- Findings from this large sample of mother–infant dyads indicate that infants who are exclusively breastfed for at least 1 month have significantly lower fat‐free mass and greater body fat percent at 6 months of age compared with formula‐fed infants.
- Additional research with stronger designs for causal inference, more precise definitions of exclusive breastfeeding, longer infant follow‐up and assessment of distribution of fat mass and fat‐free mass within an infant's body is warranted.

## 1 INTRODUCTION

‘Breastfeeding is one of the most highly effective preventive measures a mother can take to protect the health of her infant and herself’ (Benjamin, 2011; Office of the Surgeon General [US], 2011). This recommendation has underpinnings rooted in ‘obesity prevention,’ although the purported benefit is far from resolved (Cope & Allison, 2008; Gillman, 2011; Jiang & Foster, 2013; Smithers, Kramer, & Lynch, 2015). The lack of clarity about the protective effect of breastfeeding on obesity is a result of variation in study design, with most of the evidence deriving from observational studies in high‐income countries (where breastfeeding is closely linked with an important confounder—high socio‐economic status) (Horta & Victora, 2013). While randomized controlled trials would provide more causal support for the long‐term protective effect of breastfeeding on obesity, such studies are uncommon owing to the ethical issues surrounding the allocation of infants to non‐breastfeeding groups given the known short‐term benefits of this feeding mode (Martin et al., 2013a, 2013b). Further, potential confounders are often unaccounted for within observational studies. For example, studies often fail to consider non‐nutritive bioactive factors in breast milk that may be preferentially expressed in various maternal metabolic states (i.e., obesity) (Fields et al., 2017; Fields, Schneider, & Pavela, 2016; Isganaitis et al., 2019; Whitaker et al., 2017). Also, maternal body mass index (BMI) in breastfeeding mothers is often not reported or considered in these studies, thus overlooking the inherent biological heterogeneity of breast milk produced by mothers with various genotypes and phenotypes and who consume different diets (Goran, Martin, Alderete, Fujiwara, & Fields, 2017). Collectively, it is for the stated reasons that a deeper look is needed to better understand the role of early growth in infants who are breastfed (BF) or formula‐fed (FF).

Growth patterns differ between BF and FF infants, such that FF infants appear to have more rapid gains in weight‐for‐length and higher absolute weights during the first year of life (Appleton et al., 2018; Dewey, 1998; Ziegler, 2006). However, differences in infant body composition by mode of feeding are less conclusive (Gale et al., 2012; Mulol & Coutsoudis, 2017; Robinson et al., 2009), despite the relevance of early‐life adiposity accrual to later‐life obesity (Koontz, Gunzler, Presley, & Catalano, 2014). Assessment of this association is of particular interest in the first 12 months of life when the effect of infant feeding on body composition may be more prominent, and the relationship may be less confounded by other growth‐related variables (Gale et al., 2012).

In a recent systematic review and meta‐analysis of 26 studies conducted by Gale et al. (2012), fat mass percent and total fat mass were higher in BF infants at 3–4 months and at 6 months. At 12 months, however, an apparent switch occurred; fat mass at 12 months was lower in BF infants than in their FF counterparts. The differences in growth patterns between breastfeeding and formula feeding remain murky, partly because of small sample sizes and a lack of methodologic sophistication in the determinants of body composition. The primary purpose of this study was to investigate differences in anthropometric and body composition variables at 6 months as measured by dual‐energy X‐ray absorptiometry (DXA) in infants either exclusively BF or exclusively FF to at least 1 month.

## 2 METHODS

**FIGURE 1:**
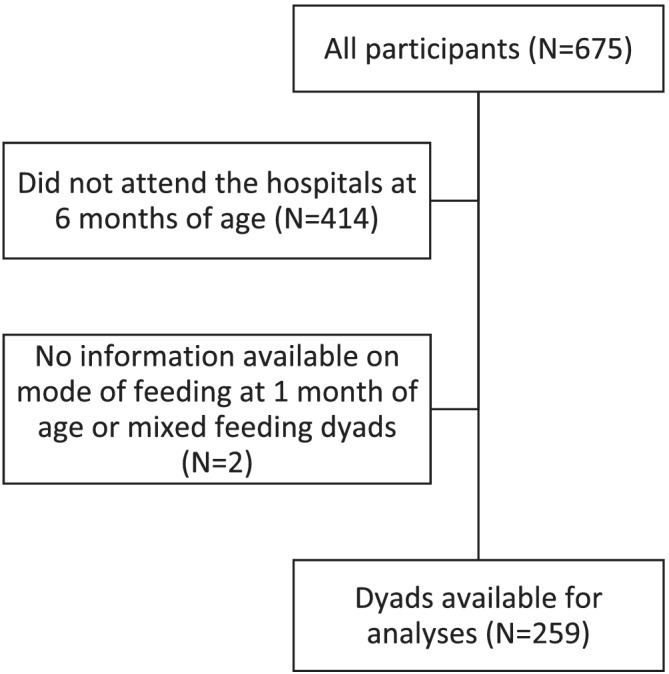
Flowchart of the selection process for the analysis

The Metabolic Research Program in the Department of Pediatrics at the Oklahoma Health Sciences campus has conducted numerous growth studies since 2008 ((Fields & Demerath, 2012; Hull, Dinger, Knehans, Thompson, & Fields, 2008; Tahir et al., 2019; Ziegler et al., 2015). A review of infants who enrolled in these studies (~750) was initially undertaken. A search was performed to include infants who visited the research centre at 6 months of age (150–210 days of age) and whose mode of feeding was known and exclusive (*n* = 675). We then restricted the study sample for the purpose of this paper to only those infants whose mode of feeding was exclusive to 1 month (*n* = 259) and had a body composition assessment by DXA in our metabolic research laboratory (Figure 1). All mothers were non‐smoking, aged 18–45 years at the time of delivery, free of diabetes with a singleton term birth. No large or small‐for‐gestational age infants were included in the study. All testing was conducted at the University of Oklahoma Health Sciences Center. The Institutional Review Board at the University of Oklahoma Health Sciences Center approved all procedures for Human Participants. Prior to testing, all mothers signed an informed consent and a Health Insurance Portability and Accountability Act authorization form.

### 2.1 Assessment of infant feeding mode

Infant feeding mode was self‐reported by the mother at 1 month upon her visit to our research/hospital facilities. Exclusive breastfeeding was defined as consuming only breast milk for the entire preceding time period with <12 oz of formula and consuming only breast milk 2 weeks before the study visit. Exclusive formula feeding was defined as consuming only formula for the entire preceding time period.

### 2.2 Anthropometric and body composition variables

#### 2.2.1 Anthropometry

Infant naked body weight was obtained using a Seca 728 scale (Seca, Hamburg, Germany) in duplicate with both measures having to be within 10 g of one another. The crown‐to‐heel length was obtained using a Seca 416 infant scale (Seca, Hamburg, Germany) in duplicate with both measures having to be within 0.1 cm of one another. If the difference was greater than those thresholds, a third measure was taken, and the two closest were averaged. Infant weight‐for‐length *z* scores (WLZ) were computed based on the World Health Organization (WHO) Child Growth Standards (WHO, 2003).

#### 2.2.2 Body composition

Infant whole body composition (total fat mass, fat‐free mass, trunk fat mass and body fat percent) was determined using DXA, specifically, a Lunar iDXAv11‐30.062 (Infant whole body analysis enCore 2007 software, GE, Fairfield, CT, USA) scanner as described previously (Fields, Demerath, Pietrobelli, & Chandler‐Laney, 2012). Study personnel placed the infant supine on the scanning bed with the infant wearing only a disposable diaper and swaddled in a hospital receiving blanket provided by the laboratory. Typically, the infant was drowsy but awake and calm during the procedure. The lights were off, and an animated movie was played on a portable DVD player outside the scanning field. No infant was re‐scanned. The same person (DAF) positioned and analysed the scans.

### 2.3 Other variable assessment

Maternal prepregnancy weight (kg), height (cm), BMI (kg/m^2^), gestational weight gain (kg) and age (years) and infant sex (female and male), birthweight (g), length (cm) and gestational age (weeks) were abstracted from medical records. Maternal/infant race (White, African American, other [including Asian American, American Indian and Mixed Race]), income (<$30,000, $30,000–$60,000, >$60,000–$90,000, >$90,000), education (high school or less, some university courses and above) and parity were self‐reported by mothers.

### 2.4 Statistical analyses

Anthropometric measurements (weight [kg], length [cm] and WLZ) and body composition measurements (total fat mass [kg], total fat‐free mass [kg], trunk fat mass [kg] and body fat percent [%]) were used as outcomes of infant growth at 6 months of age. Maternal and infant characteristics were described using means ± standard deviations and frequencies. Differences in participant characteristics by mode of infant feeding were tested using unpaired *t* tests and chi‐square tests for continuous and categorical variables, respectively. We then used a general linear model to test the association between mode of infant feeding (breastfeeding vs. formula feeding) and infant weight, length, WLZ, total fat mass, total fat‐free mass and trunk fat mass at 6 months of age. For body fat percent, we performed a median regression alternative to the general linear model given that these outcomes are proportions and tend to be non‐normally distributed (i.e., skewed), which cannot be addressed by the general linear model (Koenker, 2005). All models were adjusted for maternal age, maternal prepregnancy BMI, gestational weight gain, parity, race, infant gestational age and infant age at the visit, as these variables were associated with the exposure and/or are considered potential confounders per the literature. Of the 259 infants whose mode of infant feeding was known and who visited the research centre at 6 months, ~17.3% of outcome variables were missing. We therefore performed the analysis with multiple imputation (using 25 imputations). For imputation, we used a linear regression where all the variables used in the analysis (i.e., both outcomes and predictors) were included. Multiple imputation is ‘the process of replacing missing data with one or more specific values, and allows statistical analysis to include all participants rather than just those who do not have any missing data’ (Li, Stuart, & Allison, 2015). This procedure avoids a loss of power and the capturing of biased estimates, which can occur if subjects with missing data are excluded. All analyses were carried out on each imputed data set and combined based on Rubin's standard rules (Rubin, 1987). We used the statistical computing software R 3.4.1 for all analyses, the package ‘mice’ for imputation and ‘Zelig’ to combine the results from all imputed data. All *P* values were computed based on a two‐tailed test using .05 as significance level. The corresponding R code is available on request.

## 3 RESULTS

Among 675 total mother–infant dyads, 259 dyads had visited the research centre at ~6 months of age and had information available on mode of infant feeding at 1 month. Among these, 41 were exclusively FF, and 218 were exclusively BF at 1 month. Maternal and infant characteristics are summarized by mode of infant feeding in Tables 1 and 2, respectively. Mothers included in the study were predominantly White and highly educated. Mothers who BF were older (*P* = .002), more likely to be White (*P =* 0.011), and gained less weight during pregnancy (*P* = .009) than mothers who FF. Compared with FF infants, BF infants had a greater gestational age at birth (*P* = .013), were older at their 6‐month visit (*P* < .001) and had lower total fat‐free mass (*P* = .002), higher trunk fat mass (*P* = .032) and higher body fat percent (*P* < .001) at 6 months of age.

**TABLE 1:** Maternal characteristics by mode of infant feeding (*N* = 259)

Variable	Formula‐fed	Breastfed	*P* value^a^
	Mean (*SD*) or *N* (%)		
Total number	41	218	
Maternal age at infant birth (years)	27.0 (4.4)	29.2 (4.1)	.002*
Income			
< $30,000	5 (14.7%)	26 (14.2%)	1.000
$30,000–$60,000	11 (32.4%)	59 (32.2%)	
> $60,000–$90,000	11 (32.4%)	58 (31.7%)	
> $90,000	7 (20.6%)	40 (21.9%)	
Race			
White	32 (78.0%)	194 (89.4%)	.011*
African American	4 (9.8%)	3 (1.4%)	
Others	5 (12.2%)	20 (9.2%)	
Education			
Graduated high school or less	3 (18.8%)	16 (13.4%)	.700
Some university courses & above	13 (81.3%)	103 (86.6%)	
Height (cm)	164.8 (8.2)	165.6 (6.5)	.521
Prepregnancy weight (kg)	73.5 (19.5)	72.3 (19.3)	.738
Prepregnancy BMI (kg/m^2^)	27.0 (6.4)	26.3 (6.6)	.520
Gestational weight gain (kg)	15.9 (6.9)	12.8 (6.6)	.009*
Parity	1.8 (0.8)	2.1 (1.1)	.145

*Note*: Continuous variables are summarized as mean (*SD*) and categorical variables as frequency among the individuals who reported (column %).

^a^ Student's unpaired two‐sided *t* test for continuous variables after confirming the difference in variance was not significant using *F* test, except for maternal age at infant birth (for this variable, we used Welch's unpaired two‐sided *t* test because the difference in variance was significant); Fisher's exact test for categorical variables.

* *P* < .05.

**TABLE 2:** Infant characteristics by mode of infant feeding (*N* = 259)

Variable	Formula‐fed	Breastfed	*P* value^a^
Total number	41	218	
Sex			
Female	22 (53.7%)	96 (44%)	.306
Male	19 (46.3%)	122 (56%)	
Race/ethnicity			
White	30 (73.2%)	181 (83.8%)	.074
African American	5 (12.2%)	8 (3.7%)	
Other race/ethnicity	6 (14.6%)	27 (12.5%)	
Birth weight (gram)	3,428 (422)	3,540 (499)	.179
Birth length (cm)	50.7 (2.2)	51.5 (2.6)	.065
Gestational age (weeks)	39.0 (1.1)	39.5 (1.1)	.013*
Age at visit (days)	168.9 (4.0)	178.0 (10.8)	<.001*
Weight at 6 months of age (g)	7,469 (966)	7,544 (1000)	.659
Length at 6 months of age (cm)	66.0 (2.8)	65.9 (2.6)	.675
WLZ	0.1 (0.9)	0.2 (1.1)	.541
Total fat mass at 6 months of age (g)	2,370 (450)	2,554 (570)	.069
Total 6 fat‐free months mass at 6 months of age (g)	5,470 (600)	5,105 (640)	.002*
Trunk fat mass at 6 months of age (g)	715 (197)	808 (244)	.032*
Body fat % at 6 months of age (%)	30.1 (3.1)	33.1 (3.6)	<.001*

*Note*: Continuous variables are summarized as mean (*SD*) and categorical variables as frequencies (column %).

Abbreviation: WLZ, weight‐for‐length *z* scores.

^a^ Student's unpaired two‐sided *t* test for continuous variables after confirming the difference in variance was not significant using *F* test; Fisher's exact test for categorical variables.

* *P* < .05.

After adjustment for covariates, mode of infant feeding was unrelated to weight, length or WLZ at 6 months (Table 3). In contrast, total fat‐free mass at 6 months was significantly lower (*β* = −372 g [SE = 125, *P* = .003]), and body fat percent was significantly greater (*β* = 3.30% [SE = 0.91, *P* < .001]) in BF infants than in FF infants (Table 4). There were no other statistically significant associations between mode of infant feeding and body composition variables.

**TABLE 3:** Covariate‐adjusted association of mode of infant feeding with anthropometric measures at age 6 months

	Weight (g)		Length (cm)		WLZ	
Variable	Estimate (SE)	*P* value	Estimate (SE)	*P* value	Estimate (SE)	*P* value
1‐month formula‐fed	Reference		Reference		Reference	
1‐month breastfed	−116(190)	.540	−0.56(0.48)	.245	−0.08(0.21)	.705
White	Reference		Reference		Reference	
African American	−157 (293)	.593	0.42 (0.74)	.570	−0.41 (0.32)	.205
Other race/ethnicity	59 (193)	.759	0.19 (0.49)	.696	−0.02 (0.21)	.916
Mother's age (years)	2 (17)	.915	0.01 (0.04)	.897	0.01 (0.02)	.775
Prepregnancy BMI (kg/m^2^)	11 (11)	.309	0.02 (0.03)	.545	0.01 (0.01)	.305
Gestational age (weeks)	153 (56)	.006*	0.48 (0.14)	.001*	0.06 (0.06)	.304
Infant age at visit (days)	18 (6)	.005*	0.05 (0.02)	.003*	0.01 (0.01)	.149
Gestational weight gain (kg)	28 (11)	.009*	0.06 (0.03)	.028	0.02 (0.01)	.144
Parity	4 (63)	.951	−0.16 (0.16)	.323	0.06 (0.07)	.403

*Note*: Parameter estimates are from a single model containing all the listed variables.

Abbreviations: BMI, body mass index; SE, standard error; WLZ, weight‐for‐length *z* scores.

* *P* < .05.

**TABLE 4:** Covariate‐adjusted association of mode of infant feeding with body composition outcomes at age 6 months

	Total fat mass (gram)		Total fat‐free mass (g)		Trunk fat mass (g)		Percent fat (%)^a^	
Variable	Estimate (SE)	*P* value	Estimate (SE)	*P* value	Estimate (SE)	*P* value	Estimate (SE)	*P* value
1‐month formula‐fed	Reference		Reference		Reference		Reference	
1‐month breastfed	119 (117)	.310	−372 (125)	.003*	59 (50)	.239	3.30 (0.91)	<.001*
White	Reference		Reference		Reference		Reference	
African American	−213 (185)	.253	−152 (197)	.442	−116 (80)	.146	−1.87 (2.62)	.477
Other race/ethnicity	37 (123)	.762	95 (131)	.468	7 (53)	.896	−0.31 (0.89)	.728
Maternal age (years)	5(11)	.663	−10 (12)	.382	1 (5)	.857	0.03 (0.08)	.724
Prepregnancy BMI (kg/m^2^)	4 (7)	.542	5 (7)	0.513	2 (3)	.536	−0.02 (0.04)	.712
Gestational age (weeks)	18 (37)	.635	87 (40)	.030*	17 (16)	.293	−0.12 (0.23)	.607
Infant age at visit (days)	8 (4)	.041	7 (4)	.110	3 (2)	.094	0.01 (0.03)	.608
Gestational weight gain (kg)	14 (7)	.040*	13 (7)	.064	4 (3)	.135	0.02 (0.04)	.644
Parity	8 (41)	.846	6 (44)	.889	9 (18)	.631	0.16 (0.22)	.483

Abbreviations: BMI, body mass index; SE, standard error.

^a^ Median regression was applied for this outcome. Parameter estimates are from a single model containing all the listed variables.

* *P* < .05.

## 4 DISCUSSION

In this large, well‐defined group of mother–infant dyads, we tested differences in anthropometry and body composition at 6 months of age among infants who were exclusively BF or exclusively FF at 1 month. After controlling for covariates, our primary finding was that fat‐free mass was lower, and body fat percent was greater in BF infants at 6 months of age. These observations suggest that the lower risk of obesity that some have observed among BF compared with formula–fed infants may not be due to tracking of fat mass from early infancy.

The American Academy of Pediatrics recommends exclusive breastfeeding for 6 months (Section on Breastfeeding, 2012). Breast milk is preferred as the sole source of nutrition during early life to meet the needs of a growing and developing infant (Ballard & Morrow, 2013; Martin, Ling, & Blackburn, 2016). Specifically, human milk contains both nutrients and bioactive factors that protect against pathogens and infection, stimulate inflammatory responses, promote cell growth and bacterial colonization, regulate appetite and energy conversion and inhibit adipogenesis (Ballard & Morrow, 2013). As such, breastfeeding confers considerable benefits to the infant (Dieterich, Felice, O'Sullivan, & Rasmussen, 2013) and is associated with lower risk for obesity in infancy as evidenced by lower BMI and WLZ during the first year of life (Oddy et al., 2014; Shinn, Tangney, Busche, Sharp, & Mullen, 2018).

Despite the postulated causal role of breastfeeding in obesity prevention (Spatz, 2014), the results of recent studies examining the impact of breastfeeding versus formula feeding on body composition have been contrary to expectations (Gale et al., 2012). Our results are in line with many of these studies (Bell, Wagner, Feldman, Shypailo, & Belfort, 2017; Breij et al., 2017; Gale et al., 2012). For example, in a systematic review and meta‐analysis of studies that examined body composition in relation to breastfeeding or formula feeding, Gale et al. (2012) reported significantly lower fat mass at 3–4 months (−0.09 kg, 95% CI [−.18, −.01]) and 6 months (−0.18 kg, 95% CI [−.34, −.01]) in FF than in BF infants. Similarly, Breij et al. (2017) reported a positive association between exclusive breastfeeding duration and the percentage of subcutaneous but not visceral fat mass among infants at 6 months. Few studies have examined the association of the recommended 6 months of exclusive breastfeeding (Section on Breastfeeding, 2012; WHO, 2003) on body composition (Gale et al., 2012). In a recent secondary analysis of data from a randomized trial of maternal vitamin D supplementation that included infants who were predominantly BF for 7 months, Bell et al. (2017) demonstrated that FF infants had greater lean mass gains from birth to 7 months compared with predominantly BF infants (mean difference: 303 g, 95% CI [137, 469]). These observations of greater fat‐free mass accrual during early life among FF infants are likely explained by the higher protein content of formula compared with breast milk (Martin, Ling, & Blackburn, 2016) and the higher circulating leptin levels among BF compared with FF infants in the first year of life (Savino, Costamagna, Prino, Oggero, & Silvestro, 2002).

It is important to note that human milk is highly variable both within mothers (e.g., by duration of lactation and time of the day) (Ballard & Morrow, 2013) and among mothers (e.g., by maternal BMI and gestational weight gain) as previously shown (Isganaitis et al., 2019; Sadr Dadres et al., 2019; Whitaker et al., 2017). Varying levels of human milk hormones, proteins and cytokines may in turn differentially influence infant growth and body composition (Alderete et al., 2015; Fields et al., 2016, 2017; Gridneva et al., 2018a, 2018b).

In our study, BF infants had approximately 0.37 kg (approximately 0.5 SD) lower fat‐free mass and 3.30% (approximately 1 SD) greater body fat percent compared with FF infants. These observed average differences between the groups were thus absolutely, but not relatively, small, and they were in accord with effect sizes reviewed by Gale et al. (2012) and similar in magnitude to those reported in a recent systematic review and meta‐analysis of differences in fat‐free mass between infants born to mothers with overweight or obesity and those born to mothers with normal BMIs (mean difference: 0.18 kg, 95% CI [−.07, .42]) (Castillo‐Laura, Santos, Quadros, & Matijasevich, 2015). These cross‐study comparisons are complicated by the range of postnatal ages examined but may indicate that infant feeding plays a pivotal role in determining body composition during early life.

Contrary to our findings of lower fat‐free mass and greater body fat percent among BF versus FF infants, we did not observe any concurrent significant differences in weight, length or WLZ by mode of infant feeding. These results differ from those of several other studies that observed lower weight‐for‐age *z* scores, WLZ and BMI trajectories at various ages throughout the first year of life among BF compared with FF infants (Oddy et al., 2014; Rebhan et al., 2009; Shinn et al., 2018). Compartment‐specific body composition measures (e.g., fat mass and fat‐free mass) more accurately reflect adiposity (Weber, Leonard, & Zemel, 2012) and may be more sensitive to the influence of early‐life nutrition on infant growth, although additional research is needed to confirm these findings in larger, more diverse cohorts.

### 4.1 Strengths and limitations

This study had numerous strengths, including the relatively large sample size (>200 infants) compared with similar studies (Gale et al., 2012). Body composition was measured using a state‐of‐the art method (DXA), which validly and reliably detects differences between fat mass and fat‐free mass (Demerath & Fields, 2014). It is important to keep in mind that the sum of the total fat and fat‐free mass versus the scale weight is typically not the same in infants and small children. This is partly due to how the scanner deals with the head (given it is composed of dense bone). A second issue is compliance. In an ideal world (high compliance), the ratio (scale wt/DXA wt) in adult populations is 0.98–0.99 (this is due to maximal compliance—i.e., no movement), whereas in infant populations, it is typically between 0.95 and 0.98. In our study, we saw a ratio of 0.953 and 0.985 between the FF and BF groups. We reviewed written study notes for study subjects and found a few of the FF infants moved (but did not exceed what we would deem an invalid scan).

Although choice of infant feeding may be confounded by important maternal socio‐economic and metabolic influences, we attempted to control for these by testing for and adjusting for differences in maternal prepregnancy BMI, gestational weight gain, age, race/ethnicity, parity and educational attainment and by limiting our sample to only women without diabetes who gave birth to full‐term infants. All tests and study visits were performed by the same staff in the same research facility. Furthermore, mode of feeding was rigorously defined; mothers were allowed to enrol only if they had the intent to exclusively feed a certain mode to 3 months (either breast or formula) and actually fed exclusively to at least 1 month. Few studies include infants who are predominantly exclusively BF or FF for 6 months (Bell et al., 2017; Gale et al., 2012), which may dilute any true impact of mode of infant feeding on infant body composition. Collectively, these factors may make the study results less susceptible to bias due to both known and unknown confounding and unclear definition of mode of feeding.

Among our limitations, the study sample was predominantly White, and there were approximately five times as many BF infants as FF infants, thus limiting generalizability to the greater U.S. population. Our study sample was relatively highly educated, such that over 80% of our study participants (in both feeding groups) listed attending some college. This is not typical for FF infants (Brown & Lee, 2013). We were also unable to assess differences in infant body composition beyond 6 months of age nor the distribution of fat mass or fat‐free mass (subcutaneous vs. visceral) by mode of infant feeding (Breij et al., 2017). These considerations are important given that body composition changes considerably during the first year of life (Toro‐Ramos, Paley, Pi‐Sunyer, & Gallagher, 2015), and the distribution of fat within the body is relevant to later‐life obesity and chronic disease risk (Shuster, Patlas, Pinthus, & Mourtzakis, 2012). Finally, we do not have complete information on infant mode of feeding or complementary feeding patterns between 1 and 6 months; therefore, it is difficult to assess body composition differences among those who were BF exclusively for prolonged periods and/or mixed‐fed and to attribute the observed differences in body composition solely to breastfeeding status at 1 month. Further, it is possible that infants who were exclusively BF at 1 month may have ceased exclusivity by 6 months, indicating misclassification bias towards the null.

## 5 CONCLUSION

In our large sample of mother–infant dyads, we showed that BF infants had lower fat‐free mass and greater body fat percent than FF infants at 6 months of age independently of measured maternal and infant covariates. These findings do not detract from the importance of exclusive breastfeeding during early infancy but rather suggest that body composition trajectories in the first 6 months of life do not necessarily explain the lifelong lower risk of obesity among BF compared with FF infants. Additional research with more precise definitions of exclusive breastfeeding in line with national and international feeding recommendations is warranted. Gaps in the literature exist with respect to whether any observed differences in body composition by mode of infant feeding (1) persist beyond the first 6 months of life, (2) vary by maternal prepregnancy weight status and (3) result in different distributions of fat mass and fat‐free mass within an infant's body. Finally, randomized studies are needed if causal relations are to be identified with confidence.

## CONFLICTS OF INTEREST

Indiana University School of Public Health has received funds or promises for same from Mead‐Johnson. In the last 12 months, DBA has received personal payments or promises for same from for‐profit organizations including: Biofortis; Gelesis; Fish & Richardson, P.C.; IKEA; Law Offices of Ronald Marron; Sage Publishing; Tomasik, Kotin & Kasserman LLC; Medpace; Nestle; WW (formerly Weight Watchers International, LLC). DBA was an unpaid member of the International Life Sciences Institute North America Board of Trustees. The other authors declare that they have no conflicts of interest.

## CONTRIBUTIONS

MJT: Data interpretation, assisted in writing first draft & editing of manuscript; KE: Data analyses/interpretation, assisted in writing & editing of manuscript; PL: Data analyses/interpretation, assisted in writing & editing of manuscript; EWD: Conceptualized study design, collected pertinent study end points, interpreted data results, assisted in writing & editing of manuscript; DBA: Data analyses/interpretation, assisted in writing & editing of manuscript; DAF: Conceptualized study design, collected pertinent study end points, interpreted data results, assisted in writing & editing of manuscript.
